# Prediction of Quality of Life in Patients With Parkinson’s Disease With and Without Excessive Daytime Sleepiness: A Longitudinal Study

**DOI:** 10.3389/fnagi.2022.846563

**Published:** 2022-04-13

**Authors:** Lixia Zhang, Yajing Chen, Xiaoniu Liang, Lan Wang, Jian Wang, Yilin Tang, Xiaodong Zhu

**Affiliations:** ^1^Department of Neurology, Taizhou Second People’s Hospital, Taizhou, China; ^2^Department of Neurology, Shanghai Ninth People’s Hospital, Shanghai Jiao Tong University School of Medicine, Shanghai, China; ^3^Department of Neurology, Huashan Hospital, Fudan University, Shanghai, China; ^4^Department of Neurology, Nanjing Drum Tower Hospital, Nanjing, China; ^5^Department of Neurology, Tianjin Medical University General Hospital, Tianjin, China

**Keywords:** Parkinson’s disease, excessive daytime sleepiness, quality of life, non-motor symptoms, motor subtype

## Abstract

**Objective:**

There is a lack of longitudinal studies that directly compare the quality of life (QoL) and investigate the impact of clinical factors on QoL across different excessive daytime sleepiness (EDS) statuses in Parkinson’s disease (PD); therefore, we aimed to compare QoL and reveal the potential heterogeneous predictors of QoL between patients with PD with and without EDS.

**Methods:**

We collected clinical data among 306 patients with PD over 2 years. EDS was assessed by the Epworth Sleepiness Scale and QoL was measured with the 39-item Parkinson’s Disease Questionnaire.

**Results:**

We found that at both baseline and follow-up, patients with PD with EDS had poorer QoL and suffered more non-motor symptoms including depression and clinical probable rapid eye movement sleep behavior disorder (cpRBD). The generalized linear mixed model analysis indicated that the major predictors of QoL in PD with EDS were the akinetic-rigid type, disease duration, and total levodopa equivalent dose, while in PD without EDS, the primary determinants of QoL were Hoehn and Yahr, Mini-Mental State Examination (MMSE), and cpRBD.

**Conclusion:**

Patients with PD with EDS presented with poorer QoL. Besides, the baseline predictors of future QoL differed between patients with PD with and without EDS. These findings remind clinicians to target specific clinical factors when attempting to improve QoL among patients with PD.

## Introduction

Parkinson’s disease (PD) is usually characterized by motor manifestations, but non-motor symptoms (NMS) are also highly prevalent in PD and even more disabling than motor impairment. Excessive daytime sleepiness (EDS), the main manifestation of daytime sleep disturbance, has been reported to affect about one-third of patients with PD (Feng et al., 2021). EDS not only negatively affects social function and quality of life in patients with PD, but also increases the burden on caregivers.

Quality of life (QoL) is an important index to consider in managing PD because it concerns both the functional and emotional status of patients. The NMS, especially sleep and mood disorders, have been recently shown to exert a great effect on QoL in patients with PD (Gokcal et al., 2017; Palmeri et al., 2019). Previous studies demonstrated that patients with PD with EDS tended to have worse QoL than those without EDS (Xiang et al., 2019; Yoo et al., 2019). A recent study among patients with advanced PD with long-term disease duration showed that EDS was independently related to worse QoL after adjusting relevant factors (Sun et al., 2018). However, the impact of EDS on QoL has not been clarified in longitudinal studies. Besides, the influence of clinical factors on QoL across different EDS statuses in PD has not been addressed. Identifying the potential predictors of QoL might be helpful for clinicians to apply more precise pharmacological and non-pharmacological interventions. Therefore, the aim of our study was to compare QoL and identify the predictors of QoL between patients with PD with and without EDS.

## Materials and Methods

### Study Design and Participants

All subjects were consecutively enrolled at Huashan Hospital, Fudan University from April 2009 to December 2017. The study was approved by the Human Studies Institutional Review Board, Huashan Hospital, Fudan University. All patients provided written informed consent in accordance with the Declaration of Helsinki.

The clinical diagnosis of PD was made by two senior neurologists specializing in movement disorders based on the United kingdom PD Society Brain Bank criteria (Hughes et al., 1992). In total, 306 patients with PD without histories of stroke, epilepsy, encephalitis, traumatic brain injury, malignancies, cardiac events, or severe psychiatric illness were enrolled. The patients with PD with a family history in their first-degree relatives or have onset age younger than 50 years old were suggested to take gene tests, and those with mutation frequency were excluded. Participants were required to take a comprehensive and extensive assessment annually for 2 years at the Movement Disorder Center of Huashan Hospital.

### Clinical and Neuropsychological Assessments

Demographic and clinical data were collected. The patients with PD were divided into PD with EDS group and PD without EDS group based on the Epworth Sleepiness Scale (ESS) scores at baseline (Chen et al., 2002). The ESS scores of ≥10 indicated a diagnosis of EDS. The dosages of anti-parkinsonian drugs were converted into a total daily levodopa equivalent dose (LED) for standardization (Thobois, 2006). Motor function was evaluated using the Hoehn and Yahr (H and Y) stage and the original version of Unified Parkinson’s Disease Rating Scale (UPDRS) Part III (UPDRS-III) scores (Fahn, 1987). Patients were classified as tremor-dominant type, akinetic-rigid type, and mixed type based on the UPDRS items according to the previous study (Schiess et al., 2000). The overall NMS was determined by the non-motor symptoms questionnaire (NMSQ; Chaudhuri et al., 2006). The severity of depressive symptoms was assessed by the Geriatric Depression Rating Scale (GDS), and a score of ≥10 was regarded as depression (Ertan et al., 2005). The rapid eye movement (REM) Sleep Behavior Disorder Screening Questionnaire (RBDSQ) was used as a screening tool of clinical probable RBD (cpRBD), and a score of ≥6 was determined as cpRBD (Wang et al., 2015). Olfactory function was determined using Sniffin’s sticks Screening 12 Test (SS-12) (Pinkhardt et al., 2019). The overall cognitive function of all patients was assessed using the Mini-Mental State Examination (MMSE). A comprehensive battery of neuropsychological tests was administered by two specialized physicians to examine the following cognitive domains: executive function (Stroop Color-Word Test, SCWT; Trial Making Test-B, TMT-B), attention (Symbol Digital Modalities Test, SDMT; and Trial Making Test-A, TMT-A), visuospatial function (Clock Drawing Task, CDT), memory (Auditory Verbal Learning Test, AVLT), and language (Animal Fluency Test, AFT; Boston Naming Test, BNT) (Song et al., 2020).

In our study, QoL was measured with the 39-item Parkinson’s disease Questionnaire (PDQ-39) (Tsang et al., 2002). The PDQ-39 (or PDQ-39 subdomain) summary index (SI) was standardized from the original PDQ-39 (or PDQ-39 subdomain) scores by dividing the score by the maximum possible points and then, multiplying by 100. The PDQ-39 SI (or PDQ-39 subdomain SI) ranges from 0 to 100, with higher scores representing poorer QoL.

### Statistical Analysis

Analyses were conducted using SPSS Statistics 19. Proportions were compared using the chi-square test, and in cases where sample sizes were too small, significance tests were performed using Fisher’s exact test. The Kolmogorov-Smirnov test was used to determine the normality of continuous variable distributions. Because the two groups were not balanced in terms of sex, comparisons of continuous variables between two groups were analyzed by a multiple linear regression model for normally distributed variables, and by a generalized linear model for non-normally distributed variables.

To identify which baseline variables were associated with longitudinal variation in PDQ-39 scores, the generalized linear mixed models (GLMMs) were developed. The GLMMs allowed us to take into account the repeated measurements of PDQ-39 in the same subject, which were not independent but correlated. The baseline and follow-up of PDQ-39 were all included as the dependent variable. The associations between baseline variables (sex, age, education, BMI, disease duration, H and Y stage, UPDRS-III, akinetic-rigid type, ESS, RBDSQ, GDS, SS-12, MMSE, and LED) and PDQ-39 SI were first tested using univariate analysis, and variables with *p* < 0.2 were then included for further multivariate analyses. As a result, 12 variables (i.e., sex, age, education, disease duration, H and Y stage, UPDRS-III, akinetic-rigid type, RBDSQ, GDS, SS-12, MMSE, and LED) were examined to identify the main predictors of QoL in patients with PD without EDS, while 9 variables (i.e., sex, education, disease duration, H and Y stage, UPDRS-III, akinetic-rigid type, RBDSQ, GDS, and LED) were included to determine the major associated factors of QoL in patients with PD with EDS. Results were considered significant at *p* < 0.05.

## Results

### Baseline Demographic and Clinical Characteristics of Patients With Parkinson’s Disease With and Without Excessive Daytime Sleepiness

The baseline demographic data and clinical features of patients with PD with (*n* = 55) and without EDS (*n* = 251) were shown in Table 1. The PD with EDS group presented with a greater proportion of male patients than the PD without EDS group (76.3% vs. 55.4%, *p* = 0.004), similar to previous studies (Tholfsen et al., 2015; Zhu et al., 2016). Two groups were consistent in terms of age, age at onset, disease duration, BMI, and education level.

**TABLE 1:** Demographic and clinical characteristics at baseline.

	Total (*n* = 306)	With EDS (*n* = 55)	Without EDS (*n* = 251)	*p*-Value
**Demographics information**				
Sex, male (%)	181(59.2%)	42(76.3%)	139(55.4%)	0.004
Age, years	59.0(49.8,65.0)	60.0(49.0,65.0)	58.0(47.0,65.0)	0.293^a^
Age at onset, years	53.0(44.0,60.0)	53.0(45.0,59.0)	50.0(43.0,59.0)	0.723^a^
Disease duration, months	44.0(24.0,95.0)	72.0(36.0,107.0)	47.0(27.0,103.0)	0.145^a^
Education, years	12.0(9.0,15.0)	12.0(9.0,16.0)	12.0(9.0,15.0)	0.984^a^
BMI	22.6(20.8,25.0)	23.7(21.1,26.1)	22.5(20.8,24.8)	0.492^a^
**Motor features**				
H and Y stage	2.0(2.0,3.0)	2.0(2.0,3.0)	2.0(2.0,3.0)	0.031^a^
UPDRS-III	30.0(21.0,40.0)	32.0(25.0,43.0)	31.0(22.0,42.0)	0.556^a^
Motor subtype %				0.785
Tremor-dominant type %	26(8.5%)	4(7.3%)	22(8.8%)	
Mixed type %	29(9.5%)	4(7.3%)	25(10.0%)	
Akinetic-rigid type %	251(82.0%)	47(85.4%)	204(81.2%)	
Dyskinesia (%)	41(13.4%)	11(20.0%)	30(12.0%)	1.000
Wearing off (%)	143(46.7%)	26(47.3%)	117(46.6%)	0.929
**Non-motor features**				
NMSQ	9.0(6.0,14.0)	13.0(7.0,19.0)	9.0(6.0,14.0)	<0.001^a^
ESS	6.0(3.0,8.0)	13.0(11.0,16.0)	4.0(2.0,7.0)	<0.001^a^
RBDSQ	3.0(2.0,6.0)	5.0(3.0,8.0)	3.0(2.0,5.0)	<0.001^a^
cpRBD (%)	82(26.8%)	27(49.1%)	55(21.9%)	<0.001
GDS	10.0(5.0,16.0)	13.0(8.0,19.0)	9.0(4.0,15.0)	0.002^a^
Depression (%)	155(50.7%)	36(65.5%)	119(47.4%)	0.012
SS-12	5.0(4.0,7.0)	5.0(3.0,6.0)	5.0(4.0,7.0)	0.355^a^
MMSE	28.0(27.0,29.0)	28.0(27.0,29.0)	28.0(27.0,29.0)	0.443^a^
SCWT	47.0(44.0,49.0)	49.0(47.0,50.0)	47.0(44.0,49.0)	0.328^a^
BNT	24.0(20.0,26.0)	23.5(19.0,27.0)	24.0(20.0,26.5)	0.010^a^
SDMT	37.0(25.0,47.0)	39.0(25.0,48.0)	34.0(25.5,42.5)	0.807^a^
AVLT-delay recall	5.0(3.0,6.0)	5.0(3.0,7.0)	5.0(3.0,6.0)	0.950^a^
AVLT-total	21.0(12.0,28.0)	19.5(8.0,26.8)	20.0(8.0,25.0)	0.978^a^
TMT-A	56.5(45.0,77.3)	54.0(42.5,68.0)	55.0(45.0,73.0)	0.054^a^
TMT-B	141.0(110.0,181.5)	134.5(93.3,173.0)	139.0(106.5,180.0)	0.026^a^
AFT	16.0(12.0,19.0)	15.5(11.0,19.0)	16.0(13.0,18.0)	0.885^a^
CDT	21.0(16.0,25.0)	20.5(17.0,25.0)	22.0(17.0,25.0)	0.028^a^
**Medication information (mg)**				
Drug naive (%)	50(16.3%)	6(10.9%)	44(17.5%)	0.229
Dopamine agonists (%)	163(53.3%)	31(56.4%)	132(52.6%)	0.611
Total LED	400.0(250.0,600.0)	450.0(300.0,725.6)	375.6(250.0,600.0)	0.065^a^
LED of dopamine agonists	50.0(0,0,100.0)	50.0(0,0,100.0)	50.0(0,0,75.0)	0.066^a^
LED of levodopa ± entacapone	250.0(150.0,400.0)	300.0(150.4,525.6)	225.6(112.5,338.4)	0.037^a^
LED of MAO-B inhibitors	0.0(0.0,0.0)	0.0(0.0,0.0)	0.0(0.0,0.0)	0.854^a^
LED of amantadine	0.0(0.0,0.0)	0.0(0.0,0.0)	0.0(0.0,0.0)	0.588^a^
LED of anti-cholinergics	0.0(0.0,100.0)	0.0(0.0,100.0)	0.0(0.0,100.0)	0.741^a^

*AFT, Animal Fluency Test; AVLT, Auditory Verbal Learning Test; BMI, body mass index; BNT, Boston Naming Test; CDT, Clock Drawing Task; ESS, Epworth Sleepiness Scale; GDS, Geriatric Depression Rating Scale; H and Y, Hoehn and Yahr; LED, Levodopa Equivalent Dose; MMSE, Mini-mental Status Examination; NMSQ, Non-motor Symptoms Quest; PDQ-39, The 39-item Parkinson’s disease Questionnaire; RBDSQ, REM-sleep Behavior Disorder Screening Questionnaire; SCWT, Stroop Color-Word Test; SDMT, Symbol Digital Modalities Test; SS-12, Sniffin’s sticks Screening 12 Test; TMT, Trial Making Test; UPDRS-III, Unified Parkinson’s Disease Rating Scale Part-III.*

*^a^The comparisons of variables between PD with and with EDS were analyzed using generalized linear models.*

Regarding motor symptoms, the H and Y stage was statistically higher in patients with PD with EDS than in those without EDS, but the comparison of motor severity by UPDRS-III showed no significant difference. In the matter of motor subtypes and motor complications, the frequency of akinetic-rigid type, dyskinesia, and wearing off was similar between the two groups.

As to non-motor features, patients with PD with EDS scored remarkably higher on ESS, NMSQ, RBDSQ, and GDS. Correspondingly, the proportion of cpRBD and depression were increased in patients with PD with EDS, when compared with patients with PD without EDS. Although no significant difference was observed in MMSE, patients with PD with EDS had poorer performance on BNT, TMT-B, and CDT than patients with PD without EDS. With respect to medication use, the proportion of drug-naive and the presence of dopamine agonists use were similar between the two groups. Furthermore, the LEDs of different anti-parkinsonism drugs were compared. The patients with PD with EDS tended to have more compound levodopa (*p* = 0.037) and a marginally increased use of dopamine agonists (*p* = 0.066).

### Clinical Characteristics of Patients With Parkinson’s Disease With and Without Excessive Daytime Sleepiness at 2 Years

We compared the follow-up clinical information between the two groups (Table 2). The patients with PD with EDS had higher scores on ESS, NMSQ, RBDSQ, and GDS, while cognitive function and motor features were consistent between the two groups. The presence of dopamine agonists use was less in patients with PD with EDS than patients with PD without EDS, perhaps as a result of medication adjustment strategy (*p* = 0.007). Besides, patients with PD with EDS were prone to take higher doses of compound levodopa (*p* = 0.012) and lower doses of anticholinergics (*p* = 0.018).

**TABLE 2:** Clinical characteristics at 2-year follow-up.

	Total (*n* = 306)	With EDS (*n* = 55)	Without EDS (*n* = 251)	*p*-Value
**Motor features**				
H and Y stage	2.0 (2.0, 3.0)	2.0 (2.0, 3.0)	2.0 (2.0, 3.0)	0.318^a^
UPDRS-III	29.4 ± 11.0	30.5 ± 9.0	29.2 ± 11.4	0.501^b^
Motor subtype %				0.772
Tremor-dominant type %	33 (10.8%)	5 (9.1%)	28 (11.2%)	
Mixed type %	37 (12.1%)	8 (14.5%)	29 (11.6%)	
Akinetic-rigid type %	236 (77.1%)	42 (76.4%)	194 (77.3%)	
Dyskinesia (%)	44 (14.4%)	12 (21.8%)	32 (12.7%)	0.053
Wearing off (%)	141 (46.1%)	27 (49.1%)	114 (45.4%)	0.364
**Non-motor features**				
NMSQ	13.0 (7.0, 18.0)	15.0 (8.8, 20.5)	11.0 (6.0, 16.8)	0.013^a^
ESS	6.0 (3.0, 9.0)	11.0 (7.0, 14.0)	5.0 (2.0, 8.0)	<0.001^a^
RBDSQ	4.0 (2.0, 7.0)	6.0 (4.0, 9.0)	4.0 (2.0, 7.0)	0.002^a^
cpRBD (%)	114 (37.3%)	31 (56.4%)	83 (33.1%)	0.001
GDS	11.0 (5.0, 16.0)	13.0 (9.0, 18.0)	9.0 (4.0, 16.0)	<0.001^a^
Depression (%)	159 (52.0%)	40 (72.7%)	119 (47.4%)	<0.001
SS-12	5.0 (3.0, 7.0)	5.0 (3.0, 7.0)	5.0 (4.0, 7.0)	0.216^a^
MMSE	28.0 (26.0, 29.0)	29.0 (27.0, 29.5)	28.0 (26.3, 29.0)	0.690^a^
SCWT	47.0 (44.0, 49.0)	48.0 (45.5, 49.5)	47.0 (44.0, 49.0)	0.691^a^
BNT	24.0 (21.3, 27.0)	26.0 (22.5, 27.0)	25.0 (22.0, 27.0)	0.769^a^
SDMT	35.0 (22.5, 45.0)	37.0 (21.5, 46.8)	35.0 (22.5, 45.0)	0.384^a^
AVLT-delay recall	5.0 (3.0, 7.0)	6.0 (3.0, 8.5)	6.0 (3.0, 7.8)	0.537^a^
AVLT-total	26.0 (20.0, 33.0)	28.0 (19.5, 36.0)	26.0 (20.0, 32.0)	0.338^a^
TMT-A	58.0 (45.0, 77.5)	54.0 (40.5, 71.0)	58.0 (45.0,75.5)	0.856^a^
TMT-B	139.5 (11.0, 187.3)	130.0 (94.5, 173.0)	140.0 (113.5 188.8)	0.725^a^
AFT	15.0 (12.0, 18.0)	15.0 (12.0, 17.8)	15.0 (12.0, 18.0)	0.750^a^
CDT	23.0 (16.0, 26.0)	24.0 (17.0, 26.5)	23.0 (17.0, 26.0)	0.975^a^
**Medication information (mg)**				
Drug naive (%)	11 (3.6%)	3 (5.5%)	8 (3.2%)	0.413
Dopamine agonists (%)	193 (63.1%)	26 (47.3%)	167 (66.5%)	0.007
Total LED	400.0 (300.0, 625.0)	500.0 (312.8,725.4)	450.0 (300.0, 650.3)	0.187^a^
LED of dopamine agonists	50.0 (0,0, 100.0)	12.5 (0,0, 100.0)	50.0 (0,0, 100.0)	0.240^a^
LED of levodopa ± entacapone	300.0 (150.0, 400.0)	300.0 (212.8, 562.8)	300.0 (150.0, 437.5)	0.012^a^
LED of MAO-B inhibitors	0.0 (0.0, 0.0)	0.0 (0.0, 0.0)	0.0 (0.0, 0.0)	0.961^a^
LED of amantadine	0.0 (0.0, 0.0)	0.0 (0.0, 0.0)	0.0 (0.0, 0.0)	0.288^a^
LED of anticholinergics	0.0 (0.0, 100.0)	0.0 (0.0, 100.0)	50.0 (0.0, 100.0)	0.018^a^

*AFT, Animal Fluency Test; AVLT, Auditory Verbal Learning Test; BNT, Boston Naming Test; CDT, Clock Drawing Task; ESS, Epworth Sleepiness Scale; GDS, Geriatric Depression Rating Scale; H and Y, Hoehn and Yahr; LED, Levodopa Equivalent Dose; MMSE, Mini-mental Status Examination; NMSQ, Non-motor Symptoms Quest; PDQ-39, The 39-item Parkinson’s disease Questionnaire; RBDSQ, REM-sleep Behavior Disorder Screening Questionnaire; SCWT, Stroop Color-Word Test; SDMT: Symbol Digital Modalities Test; SS-12, Sniffin’s sticks Screening 12 Test; TMT, Trial Making Test; UPDRS-III, Unified Parkinson’s Disease Rating Scale Part-III.*

*^a^ The comparisons of variables between PD with and with EDS were analyzed using generalized linear models.*

*^b^ The comparisons of variables between PD with and with EDS were analyzed using multiple generalized linear models.*

### Longitudinal Assessment of Quality of Life in Patients With Parkinson’s Disease With and Without Excessive Daytime Sleepiness

The assessment of QoL by PDQ-39 was displayed in Table 3. Overall, patients with PD with EDS had poorer QoL both at baseline (PDQ-39 SI, 28.2 ± 16.4 vs. 18.4 ± 13.9, *p* < 0.001) and at follow-up (PDQ-39 SI, 28.6 ± 17.9 vs. 19.8 ± 15.5, *p* < 0.001). At baseline, compared with the PD without EDS group, the PD with EDS group got higher scores on almost all domains (except stigma and social support), and the three most impaired domains of PDQ-39 were cognition, bodily discomfort, and mobility. At follow-up, PDQ-39 scores were reduced in all domains in patients with PD with EDS, and the three most impaired domains were cognition, bodily discomfort, and stigma.

**TABLE 3:** Longitudinal assessment of quality of life (QoL) in patients with Parkinson’s diseases (PD) with and without excessive daytime sleepiness (EDS).

	Total (*n* = 306)	With EDS (*n* = 55)	Without EDS (*n* = 251)	*p-*Value
**At baseline**				
PDQ-39 SI	20.2 ± 14.8	28.2 ± 16.4	18.4 ± 13.9	<0.001
Mobility SI	21.4 ± 21.5	31.8 ± 22.2	19.2 ± 20.7	<0.001
Daily activity SI	16.8 ± 20.1	24.1 ± 25.0	15.2 ± 18.6	0.003
Emotional well-being SI	22.8 ± 20.4	29.8 ± 21.5	21.3 ± 19.9	0.001
Stigma SI	22.4 ± 24.5	25.8 ± 24.7	21.7 ± 24.4	0.139
Social support SI	7.2 ± 12.7	9.7 ± 16.2	6.7 ± 11.8	0.094
Cognition SI	24.8 ± 20.2	39.0 ± 21.0	21.7 ± 18.6	<0.001
Communication SI	14.7 ± 19.9	22.7 ± 23.8	12.9 ± 18.5	0.001
Bodily discomfort SI	26.9 ± 22.4	34.4 ± 23.1	25.2 ± 21.9	<0.001
**At follow-up**				
PDQ-39 SI	21.4 ± 16.3	28.6 ± 17.9	19.8 ± 15.5	<0.001
Mobility SI	22.5 ± 23.4	28.6 ± 24.4	21.1 ± 23.0	0.008
Daily activity SI	18.0 ± 21.0	27.3 ± 24.9	16.0 ± 19.7	<0.001
Emotional well-being SI	21.7 ± 20.6	27.8 ± 21.0	20.4 ± 20.4	0.003
Stigma SI	23.0 ± 23.3	29.4 ± 24.1	21.5 ± 22.9	0.006
Social support SI	9.6 ± 16.4	15.3 ± 18.8	8.4 ± 15.7	0.003
Cognition SI	26.5 ± 20.7	37.5 ± 22.5	24.1 ± 19.5	<0.001
Communication SI	19.4 ± 22.7	28.2 ± 25.6	17.5 ± 21.6	0.002
Bodily discomfort SI	28.2 ± 24.3	33.2 ± 25.0	27.1 ± 24.0	0.024

*SI, summary index.*

### Predictors of Quality of Life in Patients With Parkinson’s Disease With and Without Excessive Daytime Sleepiness

The GLMMs analysis showed that greater GDS scores were associated with poorer QoL in all groups. Besides GDS scores, the baseline H and Y stage, MMSE, ESS, and LED remained significant predictors of QoL in the PD group (Table 4). For PD with EDS group, the presence of akinetic-rigid type, disease duration, and total LED at baseline were independently associated with QoL, regardless of relevant factors. As to the PD without EDS group, H and Y stage, MMSE, and RBDSQ were related to reduced QoL.

**TABLE 4:** Predictors of QoL in PD patients with and without EDS using generalized linear mixed models.

Model	Variables	B	95% CI	*p*-Value
PD				
	H and Y	4.344	(2.714, 5.973)	<0.001
	GDS	0.886	(0.755, 1.016)	<0.001
	MMSE	–0.698	(–1.075, –0.321)	<0.001
	ESS	0.416	(0.209, 0.623)	<0.001
	LED	0.006	(0.003, 0.009)	<0.001
PD with EDS				
	Akinetic-rigid type	13.137	(4.432, 21.842)	0.003
	GDS	1.150	(0.785, 1.515)	<0.001
	Disease duration	0.046	(0.001, 0.090)	0.045
	LED	0.009	(0.003, 0.015)	0.005
PD without EDS				
	H and Y	4.403	(2.670, 6.137)	<0.001
	MMSE	–0.974	(–1.360, –0.589)	<0.001
	GDS	0.791	(0.654, 0.929)	<0.001
	RBDSQ	0.457	(0.090, 0.823)	0.015

*ESS, Epworth Sleepiness Scale; GDS, Geriatric Depression Rating Scale; H and Y, Hoehn and Yahr; LED, Levodopa Equivalent Dose; MMSE, Mini-mental Status Examination; RBDSQ, REM-sleep Behavior Disorder Screening Questionnaire; UPDRS-III, Unified Parkinson’s Disease Rating Scale Part-III.*

## Discussion

This is the first longitudinal study in China exploring the associated factors of QoL in patients with PD with and without EDS. We found that the presence of akinetic-rigid type, as well as disease duration and LED contributed to QoL in patients with PD with EDS. While in patients with PD without EDS, non-motor features including cognitive dysfunction, depression, and cpRBD play more important roles in determining QoL.

We showed that patients with PD with EDS had worse performance in depression and RBD, similar to previous reports (Zhu et al., 2016; Amara et al., 2017; Xiang et al., 2019). All these NMS can be related to neurodegeneration within the brainstem, which is responsible for controlling mood, REM atonia, and sleep-awaken rhythm (Müller and Bohnen, 2013). The expansion of Lewy pathology over time may help explain the association between EDS and other NMS among patients with PD (Abbott et al., 2019). In addition, depression and RBD may profoundly impact the quality of nighttime sleep, which can induce daytime sleepiness as well. As to the relationship between EDS and cognitive deficits, research showed discrepancies (Jester et al., 2019). Studies among *de novo* patients with PD reported no significant differences in cognitive function between patients with and without EDS (Simuni et al., 2015; Amara et al., 2017). However, in patients with PD with advanced disease stages, those suffering from EDS appeared to have cognitive decline globally and in executive function (Bjornara et al., 2014; Zhu et al., 2016). In our study, patients with PD with EDS displayed worse performance on BNT, TMT-B, and CDT at baseline, but the difference became insignificant at 2-year follow-up. More longitudinal studies are warranted to clarify the association.

One novel finding of our study is that baseline EDS severity was independently related to poor QoL over time in PD after adjusting for relevant clinical factors. The negative impact of EDS on QoL may become prominent as the disease progresses (Gallagher et al., 2010; Yoo et al., 2019). The difficulty to stay awake in activities of daily living may partly account for the association between EDS and worse QoL in patients with PD. Furthermore, the negative impact of EDS on mood, cognition, and other function could also promote a reduction in QoL (Sobreira-Neto et al., 2017). Taken together, it is of great importance for clinicians to recognize EDS early and manage EDS throughout PD.

Our results were in accordance with previous studies suggesting that NMS may play an essential role in determining QoL in PD (Muller et al., 2013; Gokcal et al., 2017). Among all kinds of NMS, depression was shown to be the most consistent predictor of reduced QoL in both PD with and without EDS groups. Since depression is often overlooked by patients and physicians in clinical practice, more attention should be paid to the identification and management of depression to achieve better QoL. In our study, cognitive impairment and cpRBD also produced negative effects on future QoL among patients with PD without EDS. Mild cognitive impairment have been reported to be related to poor QoL in PD by both cross-sectional and longitudinal studies (Lawson et al., 2014, 2016). But the impact of RBD on QoL was inconsistent in different PD research (Suzuki et al., 2013; Kim et al., 2017; Kuhlman et al., 2019) and needs further exploration. These findings suggested that interventions targeting cognitive impairment and RBD might be helpful to improve QoL in patients with PD without EDS. However, we could not exclude the possible ameliorating effect of this treatment in patients with PD with EDS, as this was an observational study.

As to patients with PD with EDS, motor features might exert a more important effect on QoL than NMS. We found that the presence of akinetic-rigid type was significantly associated with a reduction in QoL among patients with PD with EDS. This finding was in line with the predictive role of akinetic-rigid type in progressive disability in patients with PD (Post et al., 2007). The axial symptoms in akinetic-rigid type could become less responsive to pharmacotherapy with disease progression. To retain independence in activities of daily living, more comprehensive measures including physical therapy for fall prevention are highly recommended in this group (Marras et al., 2020).

Previous studies demonstrated that the akinetic-rigid type was more common in early patients with PD with EDS than those without EDS (Amara et al., 2017; Suzuki et al., 2017). Besides, the akinetic-rigid type appeared to have higher ESS scores than the tremor-dominant type, after correcting for relevant factors (Suzuki et al., 2017). The common involvement of non-dopaminergic mechanisms, especially cholinergic dysfunction in akinetic-rigid and EDS pathology, could help explain the association (Müller and Bohnen, 2013). Our study showed that akinetic-rigid type was the predominant motor subtype in PD with EDS group, but we did not observe a significant difference in motor subtypes between the two groups. Perhaps the more advanced stage of our participants may account for the discrepancies.

The LED was another critical determinant of QoL in patients with PD with EDS, which failed to predict QoL among those without EDS. Dopaminergic medications, especially dopamine agonists, were reported to reduce daytime alertness and induce somnolence (Bliwise et al., 2012; Chahine et al., 2017). But other researchers found no difference in ESS scores between different dopaminergic medication and non-dopaminergic medication (Suzuki et al., 2008). Besides, the correlation between dopamine agonist and objective sleepiness evaluated with multiple sleep latency tests was not consistent in different studies (Razmy et al., 2004; Micallef et al., 2009). Thus, studies with both objective and subjective evaluation of daytime sleepiness are needed to verify the effect of different dopamine agonists on EDS. In clinical practice, non-dopaminergic drugs or non-pharmacological interventions should be considered, especially for patients with PD with EDS. This medication strategy may account for the phenomenon that the presence of dopamine agonist use in PD with EDS group was reduced at a 2-year follow-up in our study.

The strengths of our study involve the longitudinal design and comprehensive clinical assessments. In addition, this is the first study to investigate the influence of clinical factors on QoL across positive/negative EDS statuses in PD. We acknowledge some limitations in the current study. One limitation is the short duration of follow-up. There were only slight changes in PDQ-39 scores during the 2-year follow-up and EDS is reported to be non-persistent at an early stage. A long-term follow-up would help reduce the confounding effect of time and initial medication adjustment. Another limitation is the lack of objective methods for evaluating EDS, such as polysomnography (PSG) since subjective assessments of EDS are not always consistent with objective assessment. The PSG may also serve as an effective tool to identify patients with EDS patients caused by sleep-related respiratory disorders. And there was no evaluation of nocturnal sleep quality and nocturnal sleep disturbances in our study. However, some previous studies suggested that EDS in PD was dependent on the disease itself rather than nocturnal disturbances (Suzuki et al., 2008). Lastly, the sex compositions were not balanced between the two groups. However, the higher proportion of male patients in the PD with EDS group was also reported by previous studies, and the effect of sex was taken into account when performing the comparison between groups.

## Conclusion

We concluded that the baseline predictors of future QoL differed between patients with PD with and without EDS. The presence of akinetic-rigid type was a critical predictor of reduced QoL in patients with PD with EDS, thus, comprehensive measures were recommended in such patients. As to patients with PD without EDS, more attention should be paid to NMS, including cognitive decline and RBD. The different predictors of QoL and their relationships with EDS need to be confirmed in future trials with larger sample size, long follow-up, and validated evaluation methods.

## Data Availability Statement

The original contributions presented in the study are included in the article/supplementary material, further inquiries can be directed to the corresponding author/s.

## Ethics Statement

The studies involving human participants were reviewed and approved by the Human Studies Institutional Review Board, Huashan Hospital, Fudan University. The patients/participants provided their written informed consent to participate in this study.

## Author Contributions

LZ: conceptualization, methodology, formal analysis, writing – original draft preparation, and writing – review and editing. YC: conceptualization, methodology, formal analysis, writing – original draft preparation, writing – review and editing and funding acquisition. XL and LW: data curation, validation, formal analysis, and investigation. JW: supervision, project administration, and funding acquisition. YT: writing – review and editing, supervision, project administration, and funding acquisition. XZ: supervision and project administration. All authors involved have read and approved the submitted version.

## Conflict of Interest

The authors declare that the research was conducted in the absence of any commercial or financial relationships that could be construed as a potential conflict of interest.

## Publisher’s Note

All claims expressed in this article are solely those of the authors and do not necessarily represent those of their affiliated organizations, or those of the publisher, the editors and the reviewers. Any product that may be evaluated in this article, or claim that may be made by its manufacturer, is not guaranteed or endorsed by the publisher.
